# Circulating tumour DNA dynamics during alternating chemotherapy and hormonal therapy in metastatic breast cancer: the ALERT study

**DOI:** 10.1007/s10549-024-07316-8

**Published:** 2024-04-06

**Authors:** Rebecca C. Allsopp, Qi Guo, Karen Page, Shradha Bhagani, Anna Kasim, Philip Badman, Laura Kenny, Justin Stebbing, Jacqueline A. Shaw

**Affiliations:** 1 Leicester Cancer Research Centre, Department of Genetics and Genome Biology, University of Leicester, Leicester Royal Infirmary, Robert Kilpatrick Clinical Sciences Building, Leicester, LE2 7LX UK; 2 Department of Surgery and Cancer, Imperial College, Hammersmith Campus, Du Cane Road, London, W12 0NN UK; 3 Department of Life Sciences, Anglia Ruskin University, East Road, Cambridge, CB1 1PT UK

**Keywords:** Liquid biopsy, Circulating tumour DNA, Oncomine™ Breast cfDNA Assay, Breast cancer

## Abstract

**Purpose:**

Although changes in circulating tumour DNA (ctDNA) in breast cancer are well described, the kinetics of their fluctuations has not been described over short timescales. We investigated ctDNA dynamics during alternating cycles of chemotherapy and hormonal treatment in pre-treated patients with oestrogen receptor-positive metastatic breast cancer.

**Methods:**

Patients received alternating, 9-week cycles of eribulin and aromatase inhibitors (AIs). The clinical primary endpoint, progression-free survival (PFS), was monitored at 3, 6 and 9 months; secondary endpoints, clinical benefit rate (CBR), safety and tolerability profiles, were also assessed. Importantly, ctDNA fluctuations were monitored using the Oncomine™ Breast cfDNA assay to test whether biomarkers may change rapidly between chemotherapy and aromatase inhibitor (AI) treatment in the setting of advanced breast cancer, potentially reflecting disease dynamics.

**Results:**

The median PFS was 202 days (95% CI: 135-undefined) and 235 days (95% CI: 235-undefined) at 6 and 9 months, respectively, with a 50% CBR at both 6 and 9 months. Dynamic changes in ctDNA were observed in short timescales between chemotherapy and AI treatment and support the clinical benefit (CB) seen in individual patients and, critically, appear informative of acquired resistance in real time.

**Conclusion:**

Changes in ctDNA can occur rapidly and reflect changes in patients’ clinical tumour responses (NCT02681523).

**Supplementary Information:**

The online version contains supplementary material available at 10.1007/s10549-024-07316-8.

## Background

Despite therapeutic advances, 20–30% of patients with early breast cancer still die of metastatic disease, with a 5-year relative survival of about 25% [1]. Intratumoural heterogeneity, changes in response to therapy and the development of resistance present significant clinical challenges at this stage [2].

At the time of recruitment for this study, third-generation aromatase inhibitors (AIs) were the treatment of choice for postmenopausal women with newly diagnosed metastatic oestrogen receptor (ER)-positive breast cancer, either in tamoxifen-naïve patients or those progressing on adjuvant tamoxifen. The inevitable emergence of resistance, driven by a number of mechanisms [3] and fuelled by AI ineffectiveness in a hypoxic tumour environment, presents a key challenge to overcome. Current standard of care treatment includes addition of a cyclin-dependent kinase 4/6 (CDK4/6) inhibitor in the first line as evidenced by the MONARCH-3 [4] and MONALEESA-3 trials [5, 6], or second line as evidenced by the SONIA trial [7]. Despite significant advances, patients eventually do progress on endocrine therapy.

Eribulin mesylate, a novel microtubule targeting agent (MTA), induces irreversible mitotic blockage [8] and has been shown to reverse epithelial–mesenchymal transition (EMT) and reduce cancer cell migration and invasion [9]. Of significance, it also possesses an antiangiogenic effect resulting in remodelling of abnormal tumour vasculature to a more functional microenvironment, thus eliminating inner tumour hypoxia [10]. The clinical effectiveness of eribulin was derived from two phase III randomised trials: EMBRACE (Study 305, NCT00388726) [11] and Study 301 (NCT00337103) [12]. Of challenge is the fact that eribulin has dose-limiting toxicities including bone marrow suppression, peripheral neuropathy and gastrointestinal toxicity which can limit treatment duration.

The rationale behind the ALERT study was therefore to alternate cycles of eribulin and AI therapy, capitalising on eribulin’s unique ability to improve tumour hypoxia before rechallenging with AI treatment to see if the cells become re-sensitised to hormonal blockade. Additionally, this may also provide evidence for extended use of eribulin. By serial blood sampling upon treatment change, we also monitored circulating tumour DNA (ctDNA) dynamics using the Oncomine™ cfDNA breast assay to determine whether ctDNA levels fluctuate between chemotherapy and AI treatment in this setting.

## Material and methods

### Patients and DNA samples

Eight ER + patients with LABC or MBC who had received at least one chemotherapy in the advanced setting were recruited to the study. All gave written informed consent prior to participation and were over 18 years of age. The study protocol was approved by the Riverside Research Ethics Committee (15/LO/0571) and was conducted in accordance with Good Clinical Practice Guidelines and the Declaration of Helsinki. Recruitment commenced in Nov 2015 and all 8 patients had completed the study follow-up by June 2018. Twenty ml blood was taken into K2 EDTA tubes (BD Biosciences) and processed to plasma and buffy coat within 2 h of collection and cfDNA and germline DNA were isolated, as described previously [13]. FFPE tumour DNA from 1-mm tissue cores was extracted as described previously [14].

### Treatment regime

3 × 3 weekly cycles at the recommended dose of eribulin 1.23 mg/m^2^ was administered as per standard protocol intravenously over 2–5 min on days 1 and 8 of every 21-day cycle. This was followed by 9 weeks of AI treatment and then again by 3 × 3 weekly cycles of eribulin and 9 weeks of AI treatment. Patients remained on treatment for up to 9 months or until disease progression or unacceptable toxicities, whichever was sooner. Among the 8 enrolled patients, 6 were given AI’s including anastrozole or exemestane or letrozole (detailed in results section).

### Targeted next-generation sequencing

Targeted next-generation sequencing (NGS) on 20 ng of FFPE tumour DNA, lymphocyte DNA and cfDNA samples at several timepoints during treatment was performed using the Oncomine™ Breast cfDNA v1 Assay (Thermo Fisher Scientific) as described previously [13]. Library reactions set up on the Ion Chef system were run on a 540 chip on the Ion S5 XL sequencing platform (Thermo Fisher Scientific). Alignment of sequencing raw data (hg19) was performed by the Torrent Suite Software version v 5.12. All high-confidence variant calls (those with an allele molecular coverage of ≥ 2 and Allele Mol Freq [MAF %] ≥ the limit of detection for each variant) were reviewed manually using the Integrated Genomics Viewer package (v2.3.25) by two observers.

### Statistical analysis

All efficacy data in the study were analysed based on the full analysis set (FAS) which consists of all patients who receive at least one dose of study treatment. Per-protocol analysis was performed on patients who received at least one dose of study treatment and have at least one tumour assessment. PFS is defined as the time from study enrolment to first evidence of progression. RECIST v1.1 criteria were used to assess patient response to treatment by determining tumour size, PFS and objective response rate. Patients were censored at the last follow-up date if they were lost to follow-up, withdrawn from the study or not progressed at the end of study. The primary endpoint or PFS rates at 3, 6 and 9 months and median PFS with their corresponding 95% confidence intervals were estimated by the Kaplan–Meier method. The CBR is defined as the percentage of patients whose best overall response, according to RECIST v1.1, is either a complete response (CR), partial response (PR) or stable disease (SD) for at least 6 months. The CBR and its 95% confidence interval were calculated. Safety and tolerability were assessed by adverse events (AEs) and serious adverse events (SAEs) according to the Common Terminology Criteria for Adverse Events (NCI‐CTCAE v 4.03).

## Results

**Fig. 1:**
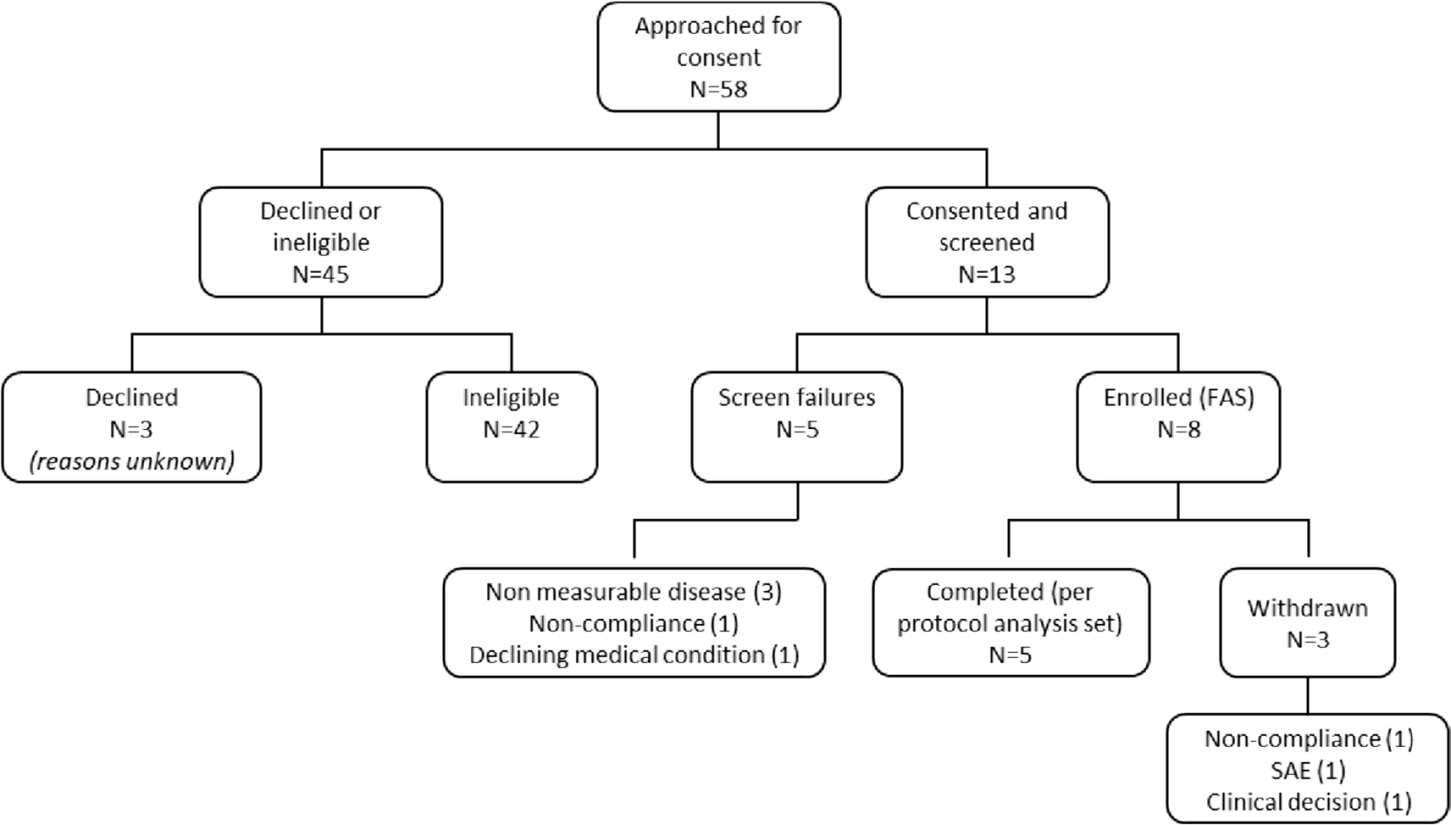
Consort diagram. A total of 58 patients were approached, 13 consented and were screened with 8 patients enrolled. Five patients completed the treatment regime while 3 withdrew due to non-compliance, SAE or clinical decision

A total of 58 patients were approached for consent. Out of these, 42 patients were ineligible, 3 declined and 13 consented. Of the 13, 8 were enrolled in the study while 5 failed to meet the inclusion criteria. Among the 8 FAS, 5 patients completed the treatment protocol while 3 discontinued due to either inability to comply, serious adverse events or investigator’s decision (Fig. 1).

The baseline characteristics of the ALERT cohort (*n* = 8) and those who had at least one follow-up tumour assessment (performed at months 3, 6 and 9) (*n* = 6) are shown in Table 1 with full tumour assessment data per patient presented in Supp Table 1.

**Table 1:** Baseline characteristics

	Full analysis set *n* = 8	Per-protocol set *n* = 6
Age in years, median (IQR)	50 (48–57)	50 (46–58)
BMI (kg/m^2^)	26.5 (20.3–31.6)	26.5 (22.5–30.0)
Ethnicity		
White	6 (75.0%)	5 (83.3%)
Turkish	1 (12.5%)	1 (16.7%)
Middle Eastern	1 (12.5%)	–
ECOG Performance status		
0	4 (50.0%)	4 (66.7%)
1	4 (50.0%)	2 (33.3%)
ER-Positive status		
Allred	3 (37.5%)	2 (33.3%)
Other	5 (62.5%)	4 (66.7%)
PgR-Positive status		
Positive	7 (87.5%)	5 (83.3%)
Negative	–	–
Unknown	1 (12.5%)	1 (16.7%)
HER2-Positive status		
0	5 (62.5%)	3 (50.0%)
1 +	1 (12.5%)	1 (16.7%)
2 +	–	–
3 +	–	–
Not done	2 (25.0%)	2 (33.3%)
Primary Tumour Type Invasive ductal carcinoma	8 (100.0%)	6 (100.0%)
Prior Chemotherapy		
Yes	8 (100.0%)	6 (100.0%)
No	–	–
Prior Radiotherapy		
Yes	8 (100.0%)	6 (100.0%)
No	–	–
Prior Endocrine		
Tamoxifen	8 (100.0%)	6 (100.0%)
Exemestane	5 (62.5%)	4 (66.7%)
Letrozole	3 (37.5%)	3 (50.0%)
Anastrozole	5 (62.5%)	3 (50.0%)
Prior surgery treatment of cancer		
Yes	8 (100.0%)	6 (100.0%)
No	–	–

Data are presented as percentages for categorical variables and median (IQR) for continuous variables

Among the 8 enrolled patients, 6 were given AI treatment (detailed in Table 2). The two patients (007 and 009) who did not receive any AI treatment withdrew from the study after 28 and 53 days. Patient 007 had inability to comply with the protocol (Supp Table 2) whereas patient 009 suffered SAEs (Supp Table 3). Both patients left the study before their month 3 follow-up. Furthermore, patient 010 left the study before the month 6 follow-up (day 78) due to poor clinical response and a decision to switch treatment. Finally, patient 002 had no more information for month 9 after developing progressive disease on month 12. Of the remaining patients, 004 and 006 completed two 9-week alternating cycles of eribulin and hormonal therapy, with patient 011 missing just the final week (week 36) of AI treatment. Patient 002 completed C1 eribulin/AI followed by C2 eribulin only (no further AI). Patient 008 completed C1 eribulin/AI followed by C2 eribulin plus 1 additional week (week 28) of AI treatment (Table 2). AI drug compliance data (the proportion of days with dose completion) were available for 5 patients whereby the median daily compliance was 100% (IQR: 95–100%) (based only on days with available treatment records) (Supp Table 2).

**Table 2:** Study medication showing the type of AI, dose and weeks of treatment for each study participant

Patient Number	Aromatase Inhibitor (AI)	Dose	Weeks of AI treatment	Total number of weeks of AI treatment
ALERT1-002	Anastrozole	1 mg	Weeks 10 to 18	9
ALERT1-004	Anastrozole	1 mg	Weeks 10 to 18,	18
			Weeks 28 to 36	
ALERT1-006	Exemestane	25 mg	Weeks 10 to 18,	18
			Weeks 28 to 36	
ALERT1-007*	n/a-	–	n/a-	–
ALERT1-008	Exemestane	25 mg	Weeks 10 to 18,	10
			Weeks 28	
ALERT1-009*	n/a-	n/a-	n/a-	n/a-
ALERT1-010*	Letrozole	2.5 mg	Weeks 10 to 12	3
ALERT1-011	Letrozole	2.5 mg	Weeks 10 to 18,	17
			Weeks 28 to 35	

Among the 8 patients recruited, 5 completed the treatment while 3 discontinued

^*^ALERT1-007, ALERT1-009 and ALERT1-010 terminated study participation after 28, 53 and 78 days, respectively

The median duration of treatment completion (from date of entry to either study completion or withdrawal from the study) for the 8 enrolled patients was 202 days (Range: 28–293 days). For the 6 patients with at least one tumour assessment, the median duration of treatment completion was 261 days (Range: 78–293 days) whereby the median tumour size changes were 27%, 15% and 2% decrease at months 3, 6 and 9, respectively (Table 3). Looking first at the sum diameters for all target lesions (Supp Table 1) by 3 months (*n* = 6), 3/6 patients had stable disease (SD) and 3/6 had a partial response (PR). By month 6 (*n* = 5), 3/5 had SD and 2/5 had progressive disease (PD). By month 9 (*n* = 4), 3/4 had SD and 1/4 PD. Focussing on the 3 patients who completed two alternating cycles of eribulin/AI, patients 006 and 011 remained stable over the 9-month duration and patient 004 remained stable for 6 months with PD by month 9. The remaining 2 patients (002 and 008) continued the treatment regime, albeit with reduced AI duration (total of 9 and 10 weeks of AI, respectively); these both showed a PR by month 3 with PD by month 6 (C1 eribulin/AI + C2 eribulin).

**Table 3:** Tumour assessment data per patient

**Full analysis set**	**Baseline**	**3 months**	**6 months**	**9 months**
Sum of the diameters (mm) for all target lesions	53.5	48	57	81.5
	(37.5–140.0)	(28.0–56.0)	(34.0–75.0)	(72.0–91.0)
	[*n* = 8]	[*n* = 6]	[*n* = 5]	[*n* = 2]
Overall response		[*n* = 6]	[*n* = 5]	[*n* = 4]
Complete response		–	–	–
Partial response		3	–	–
Stable disease		3	3	3
Progressive disease		–	2	1
Death		–	–	–
Withdrawn		–	–	–
Missing		2*	3**	4***
**Per-protocol analysis set**	**Baseline**	**3 months**	**6 months**	**9 months**
Sum of the diameters (mm) for all target lesions	53.5	48	57	81.5
	(40.0–133.0)	(28.0–56.0)	(34.0–75.0)	(72.0–91.0)
	[*n* = 6]	[*n* = 6]	[*n* = 5]	[*n* = 2]
Proportion of tumour size change from baseline		− 0.27	− 0.15	− 0.02
		(− 0.62–0.0)	(− 0.32–0.02)	(− 0.32–0.28)
		[*n* = 6]	[*n* = 5]	[*n* = 2]

Data are presented as frequency for categorical variables and median (IQR) for continuous variables

^*^ALERT1-007 and ALERT1-009 tumour assessments were not done, both terminating study participation before the 3rd month follow-up. ALERT1-007 had inability or subject failure to comply with protocol while ALERT1-009 had SAEs

^**^ALERT1-010 had no information from month 6 onwards after termination of study participation due to clinical decision

^***^ALERT1-002 had no more information for month 9 after the patient developed progressive disease on month 12. There are patients with lesions that are no longer measurable (NLM). However, these patients still have categorical overall tumour assessment data which are summarised above. ALERT1-008 was evaluated with progressive disease on month 6 and later on was classified with an overall stable disease on month 9. The patient had lesions which are NLM

The median PFS at month 3 cannot be calculated because no patient experienced disease progression at this follow-up time. At month 6, the observed median PFS was 202 days (95%CI: 135-undefined), while at month 9, the median PFS was 235 days (95%CI: 235-undefined) (Table 4). The 95% CI cannot be accurately computed due to the small sample size and limited number of events. Notably, the 3 patients reporting the longest PFS (004, 006 and 011, Table 4) were the 3 patients who received 2 alternating cycles of eribulin/AI treatments, followed by patients 008 and 002 who continued the treatment regime with reduced AI duration (Table 2). Overall, CB was reported in 4 out of 8 (50%) patients (004, 006, 008 and 011), these being 4 of the 5 per-protocol analysis set (Table 4).

**Table 4:** Clinical benefit of enrolled patients by 9 months

Patient Number	Clinical benefit	Progression-free survival (days)
ALERT1-002	Without CB	135
ALERT1-004	With CB	235
ALERT1-006	With CB	271
ALERT1-007*	Without CB	28
ALERT1-008	With CB	202
ALERT1-009*	Without CB	53
ALERT1-010*	Without CB	78
ALERT1-011	With CB	263

^*^ALERT1-007 had inability or subject failure to comply with protocol while ALERT1-009 had serious adverse events. Participation of both patients in the study got terminated before the 3rd month follow-up. ALERT1-010 had no information from month 6 onwards after termination of study participation due to clinical decision

Safety data were reported among all the patients (*n* = 8) who received at least one dose of study treatment (Table 5). In total, there were 121 adverse events (AEs) recorded from all patients, of which 10% were Grade 3 AEs and no Grade 4 and 5 AEs. Almost all (94%) of observed AEs were not serious and 86% are expected events. In terms of relationship with the study drug, 15% of the AEs are classified as ‘definitely related’, 13% are ‘probably related’ and 25% are ‘possibly related’. There were 8 serious adverse events (SAEs) observed from 5 patients during the study period (Supp Table 3). Patient 007 experienced neutropenic sepsis, classified as ‘definitely related’ to the study medication and also mucositis, deemed probably related. Patient 009 experienced dizziness and confusion, probably related to the study. Patient 010 experienced haematemesis, possibly related to the study. The 4 remaining SAEs, including hypercalcemia (patient 002), dyspnoea (patient 008), respiratory distress syndrome, confusion and multi-foci acute ischemia (patient 009) were classified as unlikely or not related to the study (Supp Table 3).

**Table 5:** Summary of adverse events^a^

Severity	No. of events *n* = 121	No. of subjects^a^ *n* = 8
Grade 1—Mild	71 (58.7%)	8
Grade 2—Moderate	38 (31.4%)	7
Grade 3—Severe	12 (9.92%)	5
AE classification		
Not serious	114 (94.2%)	8
Serious	7 (5.8%)	5
AE expectedness		
Expected	104 (86.0%)	8
Unexpected	17 (14.0%)	7
Relation to study		
Definitely	18 (14.9%)	6
Probably	16 (13.2%)	5
Possibly	30 (24.8%)	7
Unlikely	23 (19.0%)	7
Not related	34 (28.1%)	8
AE Frequency		
Continuous	36 (29.8%)	8
Frequent	19 (15.7%)	6
Intermittent	33 (27.3%)	7
Single Episode	18 (14.9%)	7
Unknown	15 (12.4%)	3

^a^A patient may have several AEs

### Total plasma cfDNA and ctDNA dynamics

The concentration of total plasma cell-free DNA (cfDNA) at screening and 9-week intervals thereafter (*n* = 24) was monitored (Supp Table 4). At the point of screening, the median cfDNA concentration of the 8 patients was 1.1 ng/ul (range 352 pg/ul to 13.8 ng/ul). For patients who received 2 alternating cycles of eribulin/AI treatment (004, 006 and 011), cfDNA concentrations showed little fluctuation from screening level for patient 004 (consistent with CB, Table 4), a fivefold increase at end of treatment (EoT) above screening level for patient 006 (consistent with overall CB) (Table 4) but inconsistent with the SD indicated at 9 months (Supp Table 1) and finally little fluctuation from screening in the week 9 and 18 samples for patient 11, consistent with the SD indicated at month 3 (Supp Table 1). Of the remaining patients, longitudinal cfDNA samples were collected for patients 002 and 008. Patient 002 cfDNA levels decreased post C1 eribulin, increased post C1 AI therapy and decreased again upon C2 eribulin, consistent with no overall CB (Table 4). Patient 008 presented with a high level of cfDNA at screening, a significant decrease (89% that of screening) by week 9 (post C1 Eribulin) with further decrease (91% that of screening) by week 18 (post AI) and again by week 36 (post C2 eribulin plus week 28 AI only) before increasing at EoT, consistent with the CB reported in Table 4.

**Fig. 2:**
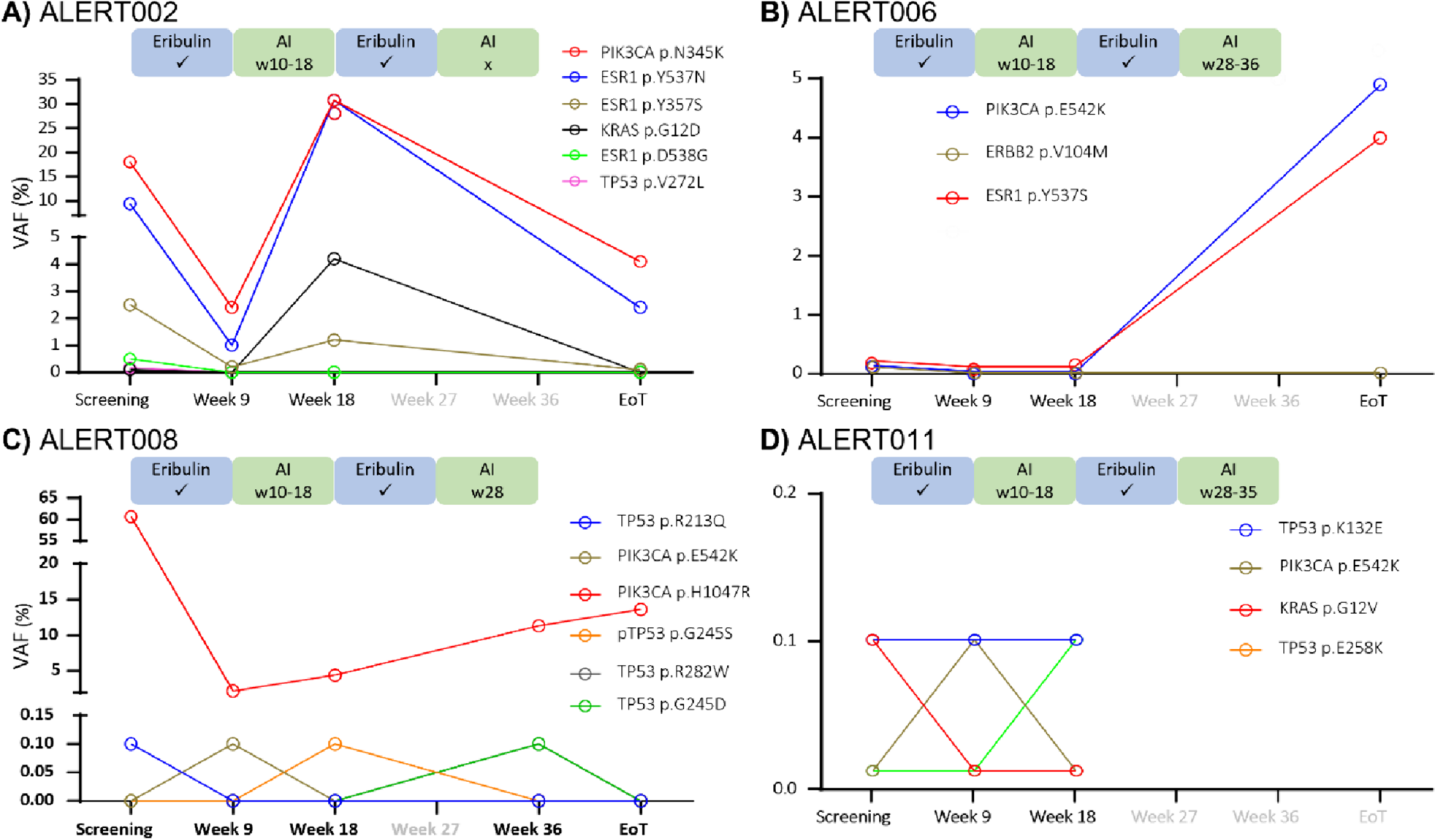
Oncomine™ cfDNA Breast Assay analysis of longitudinally collected cfDNA samples from patients (**A**) 002, (**B**) 006, (**C**) 008 and (**D**) 011. Treatment received, including the weeks of AI treatment is show for each patient. Time points at which cfDNA samples were not collected are indicated in grey font on the x-axis

Oncomine™ Breast cfDNA assay analysis was carried out in all 24 cfDNA samples for detection of ctDNA and compared with matched genomic DNA samples and FFPE tumour DNA (available for 6 patients). Analysis detected SNVs in breast cancer driver genes in all 6 FFPE tumour DNA samples; 5 patients had a *PIK3CA* driver mutation and concurrent *ESR1* mutation(s) and all 6 had multiple low-level polyclonal variants in *TP53* (Supp Table 5). Furthermore, SNVs were detected in the cfDNA of 4 of the 8 patients, 002, 006, 008 and 011 (Fig. 2A–D, respectively, and Supp Table 5). A single germline *TP53* mutation (p.H214Y) was detected (15% VAF) for patient 009 (Supp Table 6).

Concerning plasma cfDNA, patients 006 and 011 (Fig. 2B, D, respectively, and Supp Table 6) each completed 2 cycles of alternating eribulin/AI treatment. For patient 006, 3 mutations were detected in plasma cfDNA at screening (*ESR1* *p*.*Y537S* 0.18%, *PIK3CA p.E542K* at 0.12% and *ERBB2 p.V104M* 0.11%). *ESR1* *p*.*Y537S* persisted but reduced to 0.08% on switch to AI and the other 2 mutations resolved. This pattern remained stable following 9 weeks of AI, consistent with CB (Table 4). However, after switching back to eribulin, *p*.*Y537S* and *p*.*E542K* significantly increased to 4% and 4.9% VAF, respectively, in the EoT samples (Supp Table 6) indicative of progression.

For patient 011, *TP53 p.K132E* was detected at low levels (< 0.1%) at screening, 9 weeks (post C1 eribulin) and 18 weeks (post C1 AI) with no significant change in VAF. Other variants (*KRAS p.G12V, PIK3CA p.E542K* and *TP53 p.E258K*) were detected at low levels, but each in just a single sample. *ESR1* (*p*.*V392I*, 0.23% VAF) and *ERBB2* (*p*.*V104M*, 0.59% VAF) mutations detected in the FFPE tumour were undetected in plasma suggesting that these were subclonal mutations not detectable in ctDNA.

Two other patients (002 and 008) had ctDNA detected (Fig. 2A, C, respectively, and Supp Table 5), each completed one full cycle of eribulin/AI (Table 2), with patient 008 receiving additional AI treatment for one week only (week 28). Patient 002 had 6 SNVs detected in ctDNA at screening (*ESR1 p.Y537N* 9.4%, *ESR1 p.Y357S* 2.5% and *ESR1 p.D538G* 0.5%, *PIK3CA p.N345K* 18%, *KRAS p.G12D* 0.11% and *TP53 p.V272L* 0.17%). By week 9 (post C1 eribulin), 3 variants had resolved and 3 were still detected but at a lower VAF (*PIK3CA p.N345K* at 2.4%, *ESR1 p.Y357N* at 1%, and *ESR1 p.Y357S* at 0.2%) suggesting response to treatment. By week 18 (post 9 weeks AI), the VAF had increased significantly to 30.8% for both *PIK3CA p.N345K* and *ESR1 p.Y537N*; *ESR1p.Y357S* also increased to 1.2% and *KRAS p.G12D* reappeared. By the EoT sample (post C2 eribulin but no further AI), detectable ctDNA had reduced significantly to 4.1% (*PIK3CA p.N345K*), 2.4% (*ESR1 p.Y537N*) and 0.1% (*ESR1 p.Y537S*) (Supp Table 5).

Patient 008 had high ctDNA levels detected at screening (*PIK3CA p.H1047R,* 60.7% VAF) with a low frequency putative subclonal *T53 p.R213Q* variant at 0.08% VAF. The *PIK3CA p.H1047R* VAF reduced to 2.2% by week 9 (post C1 eribulin); *T53 p.R213Q* resolved, but a second putative subclonal *PIK3CA p.E524K* mutation was detected at 0.05% VAF, which was then undetected at week 18. However, *PIK3CA p.H1047R* VAF increased to 4.4% by week 18 (post C1 AI), 13.6% by week 36 (post C2 eribulin plus week 28 AI) and stabilised at 13.6% by EoT (Supp Table 5).

## Discussion

At the time of this study, postmenopausal MBC patients received first-line third-generation AI treatment until progression, upon which switching to systemic chemotherapy regimens. In an effort to prolong the effectiveness of AI therapy, patients received alternating cycles of eribulin/AI, aiming to re-sensitise cells to hormonal blockade by reducing tumour-associated hypoxia. Recent work by Goto *et al.* [15] demonstrated exactly this, showing that eribulin can induced the re-expression of ER in hypoxia-resistant breast cancer cells and in vivo xenograft models. Furthermore, by alternating treatments in this way, it is possible to provide a break to the toxicities associated with eribulin, potentially permitting the duration of its use to be extended.

A total of 8 patients were enrolled in this pilot study, with at least one follow-up tumour assessment carried out for 6 out of the 8 patients, 5 of which completed C1 alternating eribulin/AI and proceeded to C2 while 3 discontinued due to either inability to comply with the study protocol, investigator’s decision or a SAE (neutropenic sepsis; occurring in < 5% patients treated with eribulin [16]). Four out of these 5 patients (004, 006, 008 and 011) demonstrated CB, with PFS > 200 days.

Based on our previous work, simple measurement of total cfDNA levels is a good predictor of response, OS and PFS in patients with MBC [17]. The CB reported for patients 004, 006, 008 and 011 was reflected in their cfDNA levels, which remained stable for patients 004, 006 and 011 (rise is seen at EoT for patient 006) and significantly decreased (from screening level) for patient 008. Equally, patient 002 (no CB) demonstrated a consistent rise in cfDNA level, being 4 × that of screening by week 18 and 9 × higher by EoT. Of the remaining patients who withdrew from the study, only patient 010 had 1 follow-up sample analysed, showing a significant decrease in cfDNA versus screening. Overall, cfDNA quantification provided prognostic value.

Longitudinal plasma cfDNA analysis using the Oncomine™ cfDNA breast assay detected ctDNA in 4 of the 8 patients (002, 006, 008 and 011), all of whom completed C1 alternating eribulin/AI treatment. The remaining patients, including 004 (completed C1), plus 007, 009 and 010 (terminated the study during C1) showed no detectable ctDNA although patients 004 and 007 both had variants detected in the FFPE tumour tissue DNA (no FFPE was available for patients 009 and 010). Of interest, patient 009 (with no detectable ctDNA) had a single TP53 germline mutation of high VAF (15%), likely a result of clonal haematopoiesis. It is possible that use of more comprehensive mutation panel may have detected additional mutations in cfDNA samples not covered by the Oncomine assay, alternatively, inclusion of copy number analysis such as shallow whole genome sequencing on low template cfDNA [18].

An average of 4 serial time points were analysed for each ctDNA-positive patient, demonstrating ctDNA dynamics with rising or falling VAF upon treatment change. For patient 002, prominent *PIK3CA* and *ESR1* mutations followed the same trend, showing a dynamic reduction in VAF post eribulin before increasing on AI therapy, with further reduction by EoT after further eribulin only. This rapid rise seen in the final sample is indicative of disease persistence, consistent with the reported lack of CB. Both *ESR1* and *PIK3CA* mutations play a prominent role in MBC progression and endocrine resistance [19], consistent with the lack of CB of this treatment regime in this patient.

Patient 006 presented with low VAF *ESR1 p.Y537S* and *PIK3CA p.E542K* mutations at the time of screening, which remained stable during C1 eribulin/AI but increased by the EoT (following C2 eribulin/AI). Combined, these suggest molecular progression, inconsistent with the ‘stable disease’ by radiological assessment.

Patient 008 presented with a high VAF *PIK3CA p.H1047R* mutation at the point of screening at 60.75% VAF, which decreased post C1 eribulin before gradually rising throughout subsequent AI and C2 eribulin/AI treatment. Lower VAF *PIK3CA p.E542K* and polyclonal *TP53* gene mutations (*p.R213Q, G245S, pG245D*) showed small fluctuations during treatment. These data suggest that *PIK3CA p.H1047R* dynamics reflect an initial response to treatment consistent with the CB reported. However, persistence of ctDNA would suggest likely future progression.

Finally, for patient 011, ctDNA analysis at screening, week 9 (post C1 eribulin) and week 18 (post 9 weeks AI) demonstrated low-level hotspot mutations in *PIK3CA, TP53* and *KRAS* genes, consistent with CB. Of these, only *TP53 p.K132E* persisted through her treatment course.

This work demonstrates the ability to monitor ctDNA dynamics between treatments and monitor for emergence of mutations that herald treatment resistance. While the standard treatment regime has now changed to incorporate the use of CDK4/6 inhibitors plus endocrine therapy, the biomarker approach described here demonstrates the feasibility to select patients for most appropriate therapy and monitor for acquired resistance to current and new drugs. Such an approach was successfully demonstrated by the PADA-1 trial, which showed the efficacy of an early change in therapy on the basis of a rising *ESR1* gene mutations in patients with HR + HER2- metastatic breast cancer treated with AI plus CDK4/6 [20].

For the patients with detectable *PIK3CA* mutations in ctDNA (002, 006 008 and 011), a PIK3CA inhibitor may have indicated as detailed in the SOLAR-1 trail [21]. Alternatively, CDK4/6 inhibitor abemaciclib plus fulvestrant could be used regardless of *PIK3CA* or *ESR1* mutation status [22]. Furthermore, it is possible that combining eribulin with CDK4/6 inhibitor could be an effective treatment strategy in overcoming resistance to CDK4/6 inhibitors, specifically to block the escaped cells that pass the G1/S cell cycle phase irrespective of the CDK4/6 inhibitor treatment [23].

This pilot study demonstrated the ability to monitor dynamics of ctDNA between treatments in the setting of advanced breast cancer and its potential clinical utility to monitor acquired resistance and direct treatment.

### Supplementary Information

Below is the link to the electronic supplementary material.

Supplementary file1 (XLSX 19 KB)

Supplementary file2 (XLSX 15 KB)

Supplementary file3 (XLSX 18 KB)

Supplementary file4 (XLSX 19 KB)

Supplementary file5 (XLSX 20 KB)

Supplementary file6 (XLSX 19 KB)

## Data Availability

The datasets generated during and/or analysed during the current study are available from the corresponding author on reasonable request.
